# A visual risk assessment tool for acute kidney injury after intracranial aneurysm clipping surgery

**DOI:** 10.1080/0886022X.2020.1838299

**Published:** 2020-10-28

**Authors:** Pei Zhang, Chen Guan, Chenyu Li, Zhihui Zhu, Wei Zhang, Hong Luan, Bin Zhou, Xiaofei Man, Lin Che, Yanfei Wang, Long Zhao, Hui Zhang, Congjuan Luo, Yan Xu

**Affiliations:** a Department of Nephrology, The Affiliated Hospital of Qingdao University, Qingdao, China; b Beijing Anzhen Hospital, Capital Medical University, Beijing, China

**Keywords:** Intracranial aneurysm clipping surgery, acute kidney injury, nomogram, predictive model

## Abstract

**Objective:**

The aim of the study was to establish a predictive postoperative nomogram for acute kidney injury (AKI) after intracranial aneurysm clipping surgery, in order to early identify patients with high postoperative AKI risk.

**Methods:**

This is a retrospective study, which included patients who underwent intracranial aneurysm clipping surgery. Multivariate logistic regression was employed to select confound factors that associated with AKI, then incorporated into the nomogram. The predictive accuracy of the model was assessed by concordance index (C-Index).

**Results:**

A total of 365 patients after intracranial aneurysm clipping surgery were enrolled in the study eventually, of which 68 (18.63%) suffered postoperative AKI, and the incidence of stage 1, stage 2 and stage 3 were 92.65% (63/68), 5.88% (4/68), and 1.47% (1/68), respectively. Univariate logistic regression revealed that high density lipoprotein (HDL), prothrombin time (PT), estimated glomerular filtration rate (eGFR), size of aneurysm ≥10 mm, and aneurysm ruptured before surgery were associated with AKI after surgery, while multivariate logistic regression showed same results as the size of aneurysm ≥10 mm and aneurysm ruptured were independent AKI risk factors. In addition, the nomogram demonstrated a good accuracy in estimating intracranial aneurysm clipping associated AKI, as a C-Index and a bootstrap-corrected one of 0.772 and 0.737, respectively. Moreover, calibration plots showed consistency with the actual presence of AKI.

**Conclusion:**

The novel nomogram model can serve as a promising predictive tool to improve the identification of AKI among those who underwent intracranial aneurysm clipping surgery.

## Introduction

Acute kidney injury (AKI), characterized by persistent oliguria and elevated serum creatinine, is a severe postoperative complication in hospitalized patients not only associated with high mortality and cost, but also with an increased risk for the development of chronic kidney disease (CKD) and long-term cardiovascular mortality [1–4]. The incidence of AKI varies greatly according to the etiology in different clinical setting, while 40% of in-hospital AKI cases are related to surgical procedures [5]. Considering there is rare curative treatment except for renal replacement therapy up to now, prevention of postoperative AKI should be taken in advance to recognize high-risk patients and ameliorate their clinical condition [6].

As a major surgical technique to treat intracranial aneurysm, aneurysm clipping could largely prevent and repress intracranial hemorrhage by cutting off the aneurysm sac from normal circulation, which has been widely used in clinic. Diagnosis and treatment of intracranial aneurysm generally require high dose contrast agent for the imaging studies, which may cause greater risk of contrast-related kidney injury [7], but neurosurgeons seem to pay little attention to renal complications, and AKI after intracranial aneurysm clipping was rarely noticed.

Nomogram is a simple visual tool to predict the probability of a given outcome, by which researchers had applied it to predict AKI in cardiac surgery [8], nephrectomy surgery [9] in previous studies, but it has not been applied to intracranial aneurysm clipping surgery so far. Therefore, to make early diagnosis and intervention of patients with high risks of AKI after intracranial aneurysm clipping surgery, we (1) evaluated the incidence of AKI based on the Kidney Disease: Improving Global Outcomes (KDIGO) criteria; (2) analyzed the risk factors in patients who underwent intracranial aneurysm clipping surgery through multivariate logistic regression; (3) established a novel visible nomogram predictive model based on the risk factors above.

## Materials and methods

### Study population

Patients underwent intracranial aneurysm clipping surgery in the Affiliated Hospital of Qingdao University from October 2012 to October 2017 were enrolled in this retrospective observational study, and those who received continuous renal replacement therapy (CRRT) before surgery or lack of serum creatinine (Scr) level measured within 7 days after surgery were excluded.

Data used in this study was collected from our hospital information system, and there were no relevant privacy leaks of the patients, because their names were replaced with code numbers. This study was approved by the institutional review board (QDFY WZ 2018-9-13) by the Affiliated Hospital of Qingdao University to screen the serum creatinine level.

### Definition

Scr increased 0.3 mg/dL (26.5 μmol/L) within 48 h or more than 1.5-fold compared to baseline level within 7 days were diagnosed of AKI according to KDIGO 2012 guidelines. The first Scr value measured during hospitalization was defined as baseline Scr, and AKI staging was also defined according to the KDIGO guidelines. AKI was diagnosed once the participants first met the KDIGO criteria. The estimated glomerular filtration rate (eGFR) was calculated according to the Chronic Kidney Disease Epidemiology Collaboration formula (CKD-EPI) [10]. Comorbidities mentioned in this study were all defined according to the International Classification of Disease (ICD) 10th Revision.

### Statistical analyses

Indicators with more than 15% missing values were excluded from the data, while indicators with less than 15% missing values were interpolated using multiple interpolation method in *R* software with *Mice* package [11]. In addition, extreme outliers were defined as values beyond Q1 (the lower quartile)-3 × IQ (the difference between the lower quartile and the upper quartile) and were deleted to diminish bias.

We used absolute values and percentages to express categorical variables, and continuous variables were shown as the mean ± SD. Student’ s *t* test, the Chi square test, Fisher’s exact test, and the Mann–Whitney *U*-test were applied appropriately when categorical or continuous variables met the applicable conditions to calculate the differences between the two groups (AKI versus non-AKI). Then the high-risk predictors of AKI were accepted to multivariate logistic regression, in which forward stepwise regression was performed. Independent variables obtained from multivariable logistic regression were selected to construct the nomogram. As a result, odds ratios (OR) with 95% confidence intervals (95% CI) were calculated, and P value < 0.05 was considered to be statistically significant. In addition, we performed a internal validation using bootstrapping technique with 1000 resamples, and Harrell’s C statistic concordance index (the Harrell C-Index) was applied to assess the discriminatory ability of the model and to reduce overfitting bias. The statistical calculation was performed *via R* software (The *R* Foundation for Statistical Computing, https://www.r-project.org/).

## Results

### Incidence and characteristics of AKI after intracranial aneurysm clipping

**Figure 1.**
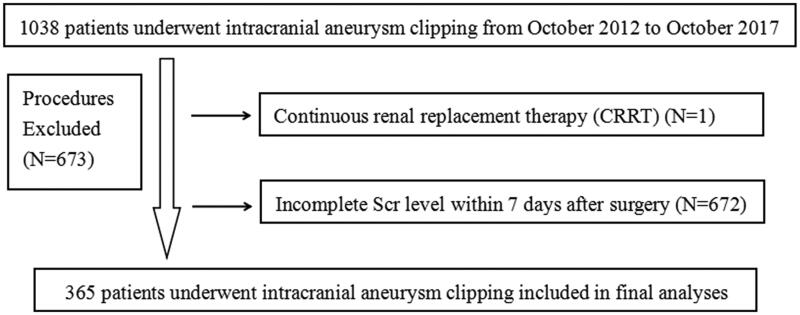
Flowchart of patient enrollment.

In this study, 1038 patients who received intracranial aneurysm clipping were included. One patient was excluded due to continuous renal replacement therapy (CRRT) before surgery, and 672 patients were excluded for incomplete Scr level within 7 days after surgery (Figure 1). At last, 365 patients were registered in the final study population, of which 68 (18.63%) suffered AKI after intracranial aneurysm clipping. Besides, the incidence of stage 1, stage 2 and stage 3 were 92.65% (63/68), 5.88% (4/68), and 1.47% (1/68), respectively, showing that patients with intracranial aneurysm clipping surgery encompassed high incidence of AKI.

Patients with AKI after intracranial aneurysm clipping showed higher incidence rates of diabetes mellitus (11.8% vs 3.8%, *p* = 0.015), but they had lower levels of estimated glomerular filtration rate (eGFR) (83.8 ± 34.7 vs 100.8 ± 18.2, *p* < 0.05). For laboratory examinations, blood glucose and several indicators in blood routine, and liver function were significantly different (*p* < 0.05) between the two groups, suggesting that glucose, white blood cell (WBC), neutrophil, albumin (ALB), and high density lipoprotein (HDL) might be related to postoperative AKI. The incidence of aneurysm ruptured before surgery (82.4% vs 69.0%, *p* < 0.05) in AKI group were different from non-AKI group, indicated that aneurysm ruptured could be a potential risk factor for postoperative AKI. (Table 1)

**Table 1.** Baseline characteristics of AKI and non-AKI.

Variable	AKI (*n* = 68)	Non-AKI (*n* = 297)	*p* Value
Demographic data			
Age (years)	60.1 ± 12.3	58.5 ± 10.5	0.299
Male (N, %)	31 (45.6%)	127 (42.8%)	0.671
BMI	24.9 ± 3.5	29.2 ± 3.1	0.334
Smoke (N, %)	17 (25.0%)	64 (21.5%)	0.537
Drink (N, %)	11 (16.2%)	49 (16.5%)	0.948
Transfusion (N, %)	6 (8.8%)	25 (8.4%)	0.914
Comorbidities			
Cerebral infarction (N, %)	6 (8.8%)	20 (6.7%)	0.546
Diabetes mellitus (N, %)	8 (11.8%)	15 (3.8%)	0.040
Coronary heart disease (N, %)	2 (2.9%)	20 (6.7%)	0.238
Hypertension (N, %)	33 (48.5%)	132 (44.4%)	0.542
Medications			
ACEI (N, %)	11 (16.2%)	28 (9.4%)	0.104
ARB (N, %)	11 (16.2%)	60 (20.2%)	0.449
β-Blocker (N, %)	31 (45.6%)	159 (53.5%)	0.237
Statin (N, %)	4 (5.9%)	26 (8.8%)	0.437
CCB (N, %)	64 (94.1%)	285 (96.0%)	0.503
Aspirin (N, %)	16 (23.5%)	78 (26.3%)	0.642
Antibiotic (N, %)	62 (91.2%)	281 (94.6%)	0.429
NSAID (N, %)	25 (36.8%)	133 (44.8%)	0.229
PPI (N, %)	65 (95.6%)	286 (96.3%)	0.784
Laboratory data			
eGFR (ml/min/1.73^2^)	83.8 ± 34.7	100.8 ± 18.2	<0.001
Scr (μmol/L)	72.3 ± 68.1	70.9 ± 23.2	0.769
Glucose (mmol/L)	7.8 ± 2.3	7.0 ± 2.5	0.015
Hb (g/L)	130.8 ± 20.0	129.9 ± 19.8	0.739
PLT (×10^9^/L)	212.9 ± 50.1	219.8 ± 63.3	0.400
RBC (×10^13^/L)	4.3 ± 0.6	4.3 ± 0.6	0.985
WBC (×10^9^/L)	11.1 ± 3.4	10.0 ± 4.0	0.040
Monocyte (×10^9^/L)	0.6 ± 0.4	0.6 ± 0.3	0.537
Neutrophil (×10^9^/L)	9.3 ± 3.3	8.0 ± 4.0	0.012
ALT (U/L)	23.1 ± 21.0	22.9 ± 23.0	0.958
AST (U/L)	21.9 ± 11.1	21.7 ± 17.5	0.934
GGT (U/L)	26.9 ± 28.5	25.4 ± 30.7	0.552
ADA (U/L)	9.3 ± 2.9	9.5 ± 3.6	0.553
TBIL (μmol/L)	15.5 ± 9.0	16.6 ± 8.7	0.368
LDH (U/L)	184.0 ± 45.4	167.2 ± 44.7	0.681
ALP (U/L)	69.8 ± 24.1	67.7 ± 20.7	0.502
TP (g/L)	62.6 ± 7.7	64.1 ± 8.1	0.137
A/G	1.4 ± 0.3	1.4 ± 0.3	0.648
ALB (g/L)	35.6 ± 4.7	37.0 ± 5.2	0.039
CHOL (mmol/L)	4.6 ± 1.0	4.9 ± 1.2	0.066
TG (mmol/L)	1.2 ± 0.6	1.2 ± 0.8	0.470
HDL (mmol/L)	1.2 ± 0.3	1.4 ± 0.4	0.005
LDL (mmol/L)	2.6 ± 0.8	2.8 ± 0.9	0.054
UA (μmol/L)	208.2 ± 118.0	206.5 ± 101.7	0.915
PT (s)	10.6 ± 1.3	10.8 ± 1.6	0.261
FIB (/L)	3.3 ± 1.0	3.3 ± 0.9	0.833
TT (s)	14.5 ± 2.5	14.2 ± 2.3	0.444
Intraoperative data			
Posterior circulation (N, %)	2 (2.9%)	14 (4.7%)	0.752
Diameter of aneurysm (N, %)	8.6 ± 7.0	8.4 ± 7.0	0.859
Diameter ≥ 10 mm (N, %)	24 (35.3%)	95 (32.0%)	0.600
Number of aneurysm (N)	1.1 ± 0.4	1.1 ± 0.3	0.611
Multiple (N, %)	7 (10.3%)	27 (9.1%)	0.758
Rupture before sugery (N, %)	56 (82.4%)	205 (69.0%)	0.028
Blood loss in operation (ml)	308.4 ± 249.0	293.7 ± 296.9	0.673
Transfusion in operation (N, %)	3 (4.4%)	14 (4.7%)	0.915
Postoperative outcomes			
CTA before discharge	22 (32.4%)	123 (41.4%)	0.168
LOS (days)	21.6 ± 18.9	17.0 ± 9.5	0.004
Death (N, %)	7 (10.3%)	4 (1.3%)	＜0.001

BMI: body mass index; NRS2002: Nutritional Risk Screening 2002; ACEI: ACE inhibitor; ARB: angiotensin receptor blocker; CCB: calcium channel blocker; NSAID: non-steroidal anti-inflammatory drugs; PPI: proton pump inhibitors; eGFR: estimated glomerular filtration rate; Scr: serum creatinine; Hb: hemoglobin; PLT: platelet; RBC: red blood cell; WBC: white blood cell; ALT: alanine aminotransferase; AST: aspartate aminotransferase; GGT: gamma-glutamyl transpeptidase; ADA: adenosine deaminase; TBIL: total bilirubin; LDH: lactate dehydrogenase; ALP: alkaline phosphatase; TP: total protein; A/G: albumin/globulin ratio; ALB: albumin; CHOL: cholesterol; TG: triglyceride; HDL: high density lipoprotein; LDL: low density lipoprotein; UA: uric acid; PT: prothrombin time; FIB: fibrinogen; TT: thrombin time; LOS: length of stay; CTA: computed tomography angiography.

Moreover, patients with AKI showed a longer length of stay during hospitalization (21.6 ± 18.9 vs 17.0 ± 9.5 days, *p* < 0.05) and higher mortality (10.3% vs 1.3%, *p* < 0.05) compared with Non-AKI patients, which means patients with AKI were related to poor prognosis after intracranial aneurysm clipping surgery.

### Predictors of AKI after intracranial aneurysm clipping

Univariate logistic regression revealed that estimated glomerular filtration rate (eGFR), high density lipoprotein (HDL), prothrombin time (PT), size of aneurysm ≥10 mm, and aneurysm ruptured before surgery were associated with AKI after surgery. To obtain the best predictors of AKI in patients with intracranial aneurysm clipping surgery, we performed multivariate logistic regression in which forward stepwise regression was used (Table 2). According to the OR in multivariate logistic regression, the high risk factors of AKI were size of aneurysm ≥10 mm (OR 2.007, CI 1.030–3.911; *p* < 0.05) and rupture (OR 3.501, CI 1.493–8.210; *p* < 0.05). These results indicated that patients with their aneurysm ≥10 mm were 1.007-fold more likely to develop AKI, and the probability of developing AKI in patients with ruptured aneurysm was 2.501 times compared with patients without ruptured aneurysm after intracranial aneurysm clipping surgery. And patients with higher eGFR, PT, and HDL were less likely to develop postoperative AKI after intracranial aneurysm clipping.

**Table 2.** Univariate logistic regression and multivariate logistic regression.

Variables	Univariate logistic regression			Multivariate logistic regression		
	β	OR (95%CI)	*p* Value	β	OR (95%CI)	*p* Value
Diabetes mellitus (N, %)	0.4585	1.582 (0.394,6.353)	0.517			
eGFR (ml/min/1.73^2^)	−0.0611	0.941 (0.920,0.962)	<0.001	−0.9969	0.369 (0.248,0.550)	<0.001
Glucose (mmol/L)	−0.0141	0.986 (0.807,1.204)	0.890			
WBC (×10^9^/L)	−0.6641	0.515 (0.224,1.180)	0.116			
Neutrophil (×10^9^/L)	0.7409	2.097 (0.953,4.613)	0.065			
ALB (g/L)	0.0781	1.081 (0.736,1.588)	0.690			
HDL (mmol/L)	−3.3222	0.039 (0.006,0.275)	0.001	−0.4945	0.609 (0.467,0.796)	<0.001
PT (s)	−0.7800	0.458 (0.298,0.704)	0.003	−0.6710	0.511 (0.305,0.857)	0.011
Rupture before sugery (N, %)	1.559	4.757 (1.563,14.480)	0.006	1.2531	3.501 (1.493,8.210)	0.004
Diameter ≥ 10 mm	1.924	6.850 (1.765,26.576)	0.005	0.6965	2.007 (1.030,3.911)	0.041

### Establishment and validation of nomogram

**Figure 2.**
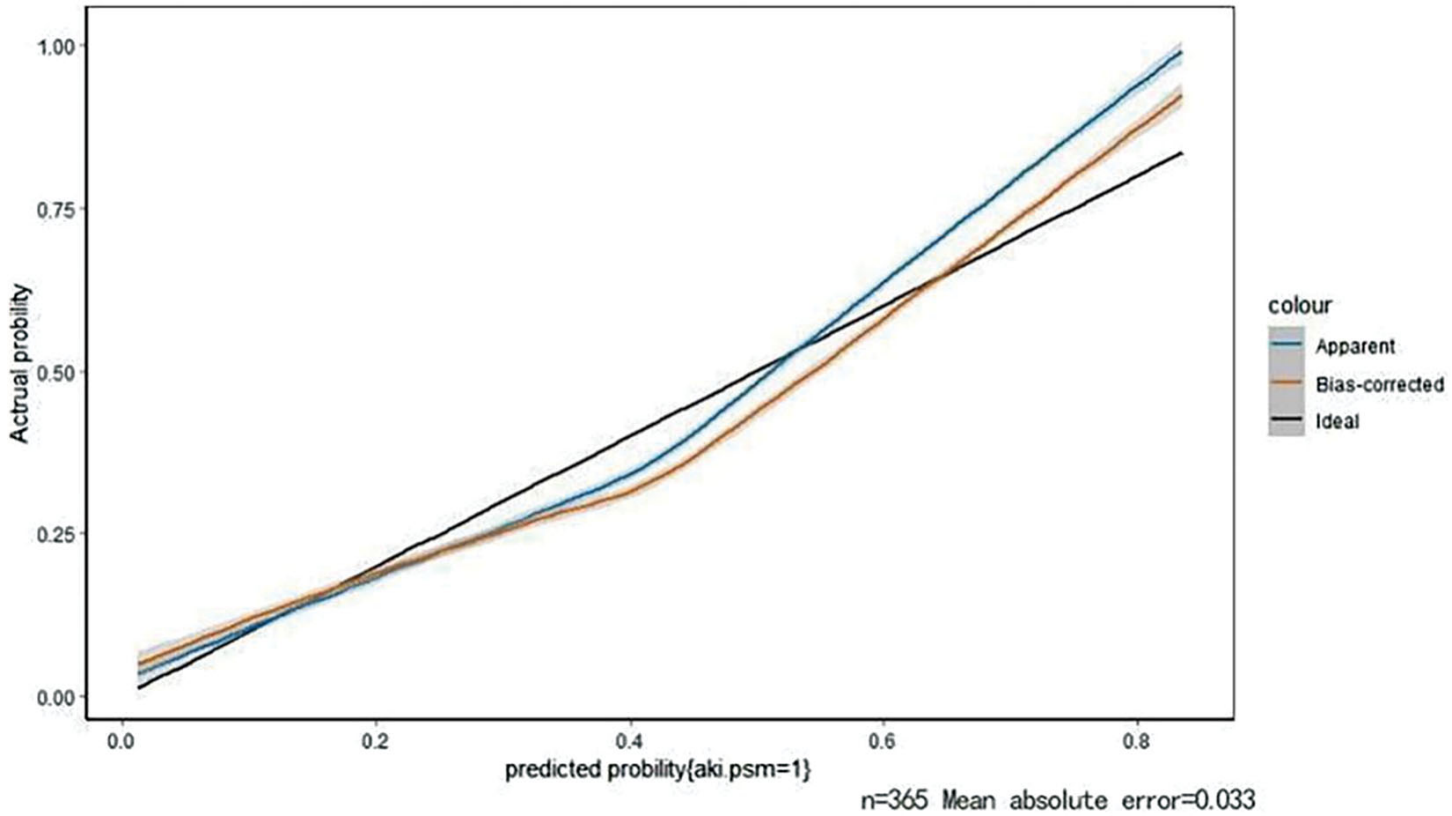
Calibration plots of internal validation. The nomogram demonstrated a good accuracy in estimating intracranial aneurysm clipping associated AKI, as a C-Index and a bootstrap-corrected one of 0.772 and 0.737, respectively.

Based on the results of multivariate logistic regression, a visual predictive nomogram of AKI patients after intracranial aneurysm clipping surgery was constructed, and each predictive factor had a relevant point which was calculated according to its regression coefficients. Then an internal validation was performed using a bootstrapping technique with 1000 resamples, and the Harrell C-Index was used to further evaluate the discrimination ability of the model and to reduce overfitting bias. The C-Index was 0.796 (SD = 0.018, 95% CI 0.795–0.797) for the primary cohort and 0.789 (SD = 0.015, 95% CI 0.788–0.790) for the bootstrapping cohort. Moreover, calibration plots showed good consistency with the actual presence of AKI (Figure 2).

**Figure 3.**
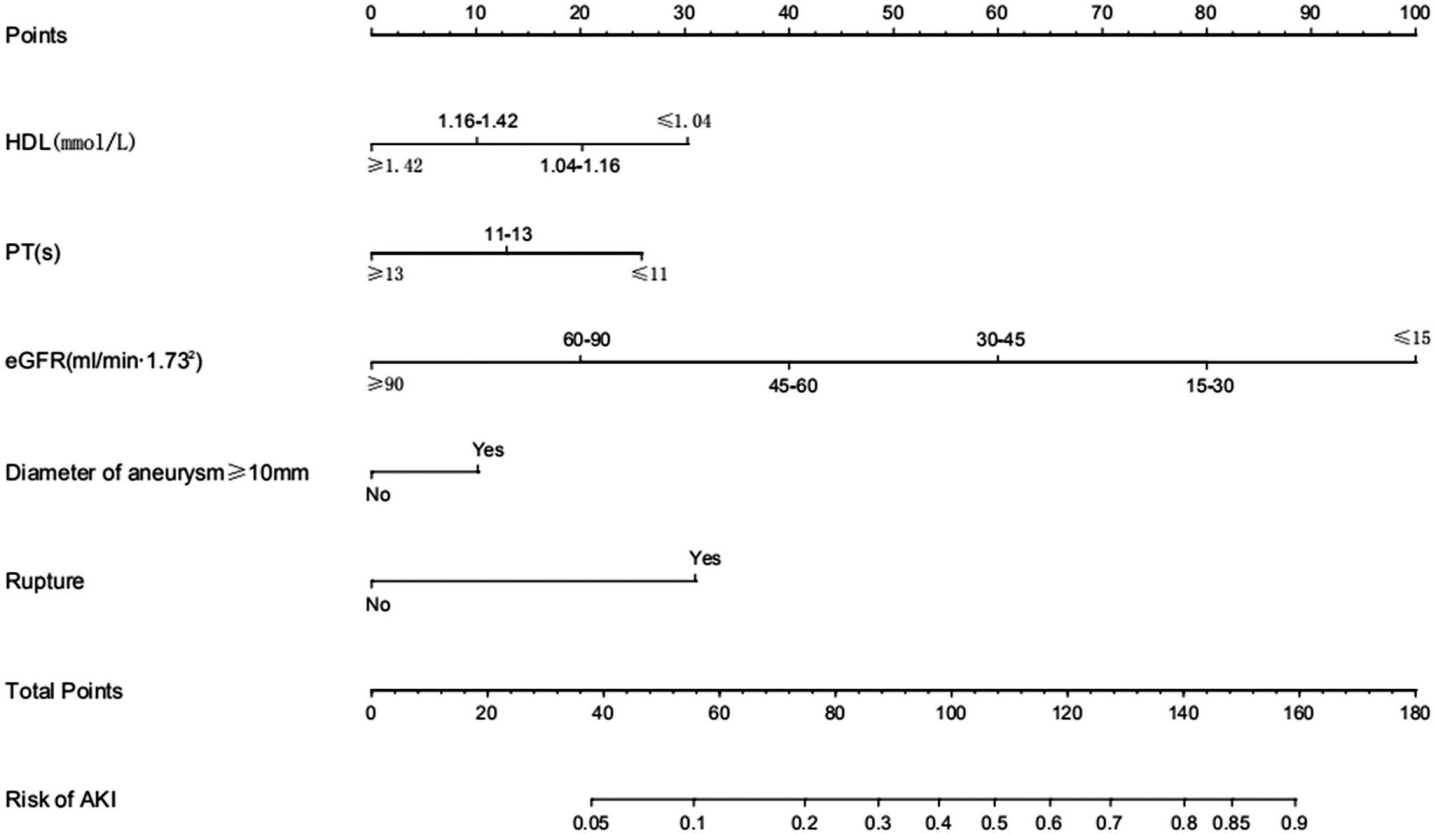
Nomogram of predictors based on multivariate regression analysis. Locate patient’s values on each axis to obtain the predicted probability of AKI after intracranial aneurysm clipping surgery. Draw a vertical line toward the ‘Points’ axis to determine the points of each variable, sum the points and locate on ‘Total Points’ axis. Draw a vertical line toward the ‘Risk of AKI’ axis to find the possibility of AKI after intracranial aneurysm clipping surgery. The predicted range of AKI was from 5% to 90%.

At last, the nomogram (Figure 3) showed that aneurysm diameter ≥10 mm and ruptured aneurysm were the largest contributors of AKI after surgery, while patients who had higher HDL, prothrombin time (PT) or eGFR were at a lower risk in AKI developing.

## Discussion

Postoperative AKI continues to be a feared consequence of surgery, it is relatively common in cardiac surgery and has short- and long-term survival implications. Previous studies demonstrated that the incidence of need for dialysis was 1.9% in AKI patients after cardiac surgery [12], and those who had a temporary recovery of renal function by the time of hospital discharge still had a high risk of death for up to 10 years [13]. Given this condition, the focus of clinicians is still on prevention and risk factor management. However, without convenient and reliable means of measuring real-time renal blood flow and the kidney histopathologic level, it is indeed confusing in detecting AKI after surgery, therefore, we established a nomogram model to serve as a promising predictive tool to improve the identification of AKI.

In this study, we found that aneurysm rupture before surgery a risk factor to predict AKI after intracranial aneurysm clipping. Rupture of aneurysm will cause subarachnoid hemorrhage and studies revealed that intrathecal secretion of inflammatory cytokines, including IL-1,IL-6 and TNF-α, was significantly increased in these patients with poor clinical outcome [14,15], they hypothesized that an overwhelming inflammatory response in the subarachnoid space plays a central part in pathogenesis of vasospasms and subsequent cerebral ischemia [15]. Studies also found that IL-1β, IL-6 and TNF-α generated by renal tubule cells after ischemic injury of kidney, could activate inflammatory cells [16], recruited inflammatory cells further release a broad range of inflammatory cytokines, which would increase further injury and fibrosis [17,18]. Whether there is a connection between ruptured aneurysm after intracranial aneurysm clipping and acute kidney injury AKI through the cytokines still need further research. Besides, patients with ruptured aneurysm may require more frequent radiographic studies and interventions with contrast agent, which may increase the risks of AKI.

Size of aneurysms as a risk factor to predict rupture of intracranial aneurysm was widely reported but still controversial. Rinkel et al. [19] revealed higher rupture risk in aneurysms larger than 10 mm compared with that were smaller than 10 mm while Cezary et al. [20] claimed just the opposite. On the other side, Beck et al. [21] found that neither height nor width of the aneurysm are significant parameters correlated with the risk of aneurysm rupture. These different results could be due to regional and ethnic differences. In this study, we found aneurysms larger than 10 mm a predictor of AKI after intracranial aneurysm clipping, since there are no relevant studies about the size of aneurysm and AKI, we assume that those larger than 10 mm are more likely to rupture in this study, thus leading to the occurrence of AKI as mentioned above.

Intriguingly, we found that ruptured aneurysm and aneurysm size ≥10 mm are risk factors for patients with intracranial clipping surgery, and blood pressure should be controlled at a relatively low level in these two conditions. However, intraoperative hypotension may substantially contribute to postoperative AKI caused by reduced renal perfusion. A retrospective analysis have reported that a mean arterial pressure less than 55 mmHg could predict adverse renal-related outcomes after noncardiac surgery [22] and the decrease of intraoperative systolic blood pressure was also associated with postoperative AKI in cardiac surgery [23]. But another analysis found that the relationship between hypotension and acute kidney injury was determined by underlying patient and procedural risk, patients with low risk demonstrated no associated increased risk of acute kidney injury across all blood pressure ranges [24]. In this condition, whether intraoperative hypotension is a confounding factor between ruptured bigger aneurysm and AKI still remains controversial, therefore, further researches are still needed in the future.

Nomogram, a single numerical estimate of the probability of an event, has shown better performance than other options of accurate estimates of the likelihood of treatment success, complications and long-term morbidity [25]. Our study is the first to establish a nomogram to predict the risk of AKI after intracranial aneurysm clipping surgery, which can provide clinical guidance for the early identification and intervention of patients with high risk.

Our study had several limitations. First of all, this was a single-center retrospective study, the stability of the model should be evaluated before applied to other population. Secondly, the diagnosis and staging of AKI based on urinary criteria according to KDIGO criteria (urine volume < 0.5 mL/kg/h for 6 h) was not adopted in this study, which may influence the diagnosis and staging of AKI, thus underestimating the occurrence of AKI. Thirdly, as a retrospective study, some indicators such as contrast agent, blood pressure etc. were not available due to the missing data, this may cause bias to some extent, thus needing further research in the future.

## Conclusion

In conclusion, HDL, PT, eGFR, aneurysm ≥10 mm, and rupture score are predictors of AKI after intracranial aneurysm clipping. Besides, we established a novel nomogram model based on the predictive factors above to predict AKI. This model can serve as a promising predictive tool to improve the identification of AKI among those who underwent intracranial aneurysm clipping surgery.
